# Regulatory effect of *Balanites aegyptiaca* ethanol extract on oxidant/antioxidant status, inflammatory cytokines, and cell apoptosis gene expression in goat abomasum experimentally infected with *Haemonchus Contortus*

**DOI:** 10.1007/s11250-024-04023-w

**Published:** 2024-07-04

**Authors:** Doaa Sedky, Tamer Helmi Abd El-Aziz, Soad Mohamed Nasr, Sekena Hassanien Abdel-Aziem, Noha Mahmoud Fahmy Hassan, Amira Hassan Mohamed, Hala Abdalla Ahmed Abou Zeina

**Affiliations:** 1 Department of Parasitology and Animal Diseases, National Research Centre, 33 Bohouth Street, Dokki, Post Box, 12622, Giza, Egypt; 2 Cell Biology Department, National Research Centre, 33 Bohouth Street, Dokki, Post Box, 12622, Giza, Egypt; 3 Department of Clinical Pathology, Faculty of Veterinary Medicine, Cairo University, Post Box, 12211, Giza, Egypt

**Keywords:** *Haemonchus Contortus*, *Balanites aegyptiaca*, Oxidant/antioxidant status, Cytokines, Cell apoptosis, Gene expression, Goats’ abomasum

## Abstract

This experiment aimed to assess the regulatory effects of treatment with *Balanites aegyptiaca* fruit ethanol extract (BA-EE) on oxidant/antioxidant status, anti-inflammatory cytokines, and cell apoptosis gene expression in the abomasum of *Haemonchus contortus*–infected goats. Twenty goat kids were assigned randomly to four equal groups: (G1) infected-untreated, (G2) uninfected-BA-EE-treated, (G3) infected-albendazole-treated, (G4) infected-BA-EE-treated. Each goat in (G1), (G3), and (G4) was orally infected with 10,000 infective third-stage larvae. In the fifth week postinfection, single doses of albendazole (5 mg/kg.BW) and BA-EE (9 g/kg.BW) were given orally. In the ninth week postinfection, the animals were slaughtered to obtain abomasum specimens. The following oxidant/antioxidant markers were determined: malondialdehyde (MDA), glutathione (GSH), glutathione-S-transferase (GST), superoxide dismutase (SOD), catalase (CAT). The mRNA gene expression of cytokines (IL-3, IL-6, IL-10, TNF-α) and cell apoptosis markers (Bax, Bcl-2) were estimated. (G1) showed significantly reduced GSH content and GST and SOD activities but a markedly increased MDA level. (G3) and (G4) revealed a markedly lower MDA level with pronouncedly elevated GSH, SOD, and GST levels. The antioxidant properties of BA-EE were superior to those of albendazole. The mRNA gene expressions of IL-3, IL-6, IL-10, TNF-α, and Bax-2 were upregulated in (G1) but downregulated in (G3) and (G4). Bcl-2 and Bcl-2/Bax ratio expression followed a reverse course in the infected and both treated groups. We conclude that BA-EE treatment has a protective role in the abomasum of *H. contortus*–infected goats. This could be attributed to its antioxidant properties and ability to reduce pro-inflammatory cytokines and cell apoptosis.

## Introduction

*Haemonchus contortus (H. contortus*), a highly pathogenic and voracious blood-feeding nematode, is a crucial gastrointestinal nematode (GIN) in small ruminants (Besier et al. 2016). The presence of this parasite causes economic losses because it reduces productivity, increases mortality rates, and requires expensive treatment (Arsenopoulos et al. 2021). *H. contortus* has a high propensity to develop resistance to anthelmintic drugs because of their overuse (Kotze and Prichard 2016; Bihaqi et al. 2020; Kaplan 2020).

During the GIN life cycle, excretory/secretory (ES) products are produced during the developmental processes to reach the adult stage (Besier et al. 2016). Many ES substances of *H. contortus*, including arginine kinase, galectins, and elongation factor 1 alpha proteins, have been found to play an essential role in modulating a wide range of biological functions, including cell activation, proliferation, adhesion, and apoptosis (Sun et al. 2007; Ehsan et al. 2017, 2020).

The inflammatory response (inflammation) is one of the most vital defense mechanisms against numerous disease conditions and is associated with possible or actual tissue damage. The principal mechanisms involved in the inflammatory reaction are intimately linked to the production of free radicals and the induction of oxidative stress induced during the aggressive activity of GINs in the gastrointestinal tract, activating multiple immune mechanisms (Gadahi et al. 2016; Estrada-Reyes et al. 2017). Excessive levels of intracellular reactive oxygen species (ROS) frequently lead to the oxidative alteration of vital biomolecules, which can hasten cell apoptosis and exacerbate the disease condition (Lennicke and Cochemé 2021; Sies et al. 2022; Wang et al. 2023). The inflammatory processes activated by haemonchosis comprise large alterations in the production of cytokines, either in the peripheral blood or in the abomasum wall (Gossner et al. 2013; Estrada-Reyes et al. 2017). The host immune reactions against GINs involve an augmentation in cytokine production and the generation of antibodies specific to the parasite (McRae et al. 2015). These reactions are linked to decreased egg production, excretion by adult worms, and decreased parasitic burden as well as regulation of the damage caused by parasite burden (Machín et al. 2021).

The emerging anthelmintic resistance, drug toxicity, and customer demand for chemical-free animal products have raised the interest in natural alternative sources of anthelmintic drugs (Besier et al. 2016; Jaheed et al. 2019). Medicinal plants contain a wide spectrum of biological functions such as immunomodulatory, antioxidant, anti-inflammatory, and antimicrobial properties that have been attributed to polyphenols (Xu et al. 2017; Yahfoufi et al. 2018). Medicinal plants may promote local immune responses and improve the secreted cytokine profile, increasing ’resistance to haemonchosis in small ruminants (Hoste et al. 2016). Among the evidence-based herbal remedies is *Balanites aegyptiaca* (L.) Del. (*B. aeg*yptiaca), often known as *desert date* in English and *lalob*, *hidjihi*, and *heglig* in Arabic. It is a member of the Zygophyllaceae family (Murthy et al. 2021). The fruits of *B. aegyptiaca* contain various kinds of compounds that possess a diverse range of pharmacological and biological activities, including antiparasitic, antioxidant, antibacterial, anti-inflammatory, and cytotoxic activities (Jaheed et al. 2019; Murthy et al. 2021; Sedky et al. 2022). Polyphenols (phenolic acids, flavonoids, and coumarins), alkaloids, steroids, saponins, and glycosides are just a few of the secondary metabolites that *B. aegyptiaca* tissues contain (Meda et al. 2011; Jaheed et al. 2019; Sedky et al. 2022). These fruits also contain minerals, major fatty acids, amino acids, and vitamins (Murthy et al. 2021). Therefore, *B. aegyptiaca* could be a source of natural antioxidant and anti-inflammatory compounds that may be extremely valuable as therapeutic agents for the prevention and treatment of various diseases (Speroni et al. 2005). In our previous study, we found *B. aegyptiaca* fruit extracts to have anthelmintic effects on some multiple-drug-resistant gastrointestinal parasites including *H. contortus*, both in vitro (Shalaby et al. 2020) and in vivo (for treatment) (Jaheed et al. 2019, 2020). *B. aegyptiaca* fruits are commonly used to purge intestinal parasites and have been found effective against *H. contortus* in goats (Jaheed et al. 2019), *Fasciola gigantica* in goats (Koko et al. 2000), *Schistosoma mansoni* in mice (Koko et al. 2005) and *Trichinella spiralis* in rats (Shalaby et al. 2010).

Based on our prior studies, *B. aegyptiaca* fruit ethanol extract (BA-EE) was abundant in phytochemicals, had a significant anthelmintic effect against *H. contortus* as evidenced by a decrease in fecal egg counts and worm burden of the parasite, and enhanced the health condition and metabolic profile of experimentally infected goats (Jaheed et al. 2019, 2020). Consequently, the purpose of this study was to investigate the regulatory influence of BA-EE treatment on oxidant/antioxidant status along with the anti-inflammatory cytokines and cell apoptotic and antiapoptotic gene expression profiles in the *H. contortus*–infected goats’ abomasa.

## Materials and methods

### *B. Aegyptiaca* fruit procurement and BA-EE preparation

*B. aegyptiaca* fruits were obtained from a local market in Aswan, Upper Egypt. Scientifically validated and voucher specimens were deposited in the Herbarium of Medicinal and Aromatic Plants Department, National Research Centre, Egypt.

*B. aegyptiaca* fruits were extracted using the approach provided by El-Newary et al. (2016). Briefly, the fresh fruits’ mesocarp of *B*. *aegyptiaca* (1 kg) was exposed to triple maceration, soaked in 4 L of 70% ethyl alcohol, and maintained in tightly sealed jars at room temperature for three weeks, during which it was stirred several times daily with a sterile glass rod. This combination has been filtered. The residue was extracted 3–5 times in the same way until a clear, colorless supernatant extraction liquid was produced. The extracted liquid was filtered and concentrated with a rotary evaporator (Heidolph 2000, Germany) under decreased pressure at 50 °C until the solvent was totally evaporated. The extract was kept at 4 °C until further use.

### Preparation of a dose BA-EE

BA-EE at a single dose of 9 g/kg.BW (body weight) was freshly suspended in 250 mL distilled water in a large bottle and drenched orally to the treated goats (Koko 2000; Albadawi 2010).

### Albendazole

The anthelmintic efficacy of BA-EE was compared with that of albendazole (Evazole®, in the form of 2.5% oral suspension), a broad-spectrum anthelmintic which was used as a reference drug for treating one of the infected groups. Albendazole was purchased from the Veterinary Division of EVA Pharma, Cairo, Egypt.

### Generation of the infective larval dose of *H. Contortus*

In order to develop infective third-stage larvae (L3) of *H. contortus*, an appropriate fecal culture was made using eggs from the collected worms (Soulsby 1982). A goat kid that tested negative for GIN infection was used as a donor, received 10,000 infective L3 in 10 ml of physiological saline solution orally, and served as a source of a monospecific L3 infection of the goats in the experimentally infected groups (Howlader et al. 1997; Jaheed et al. 2019).

### Animals and experimental approach

Twenty male Egyptian Baladi goat kids (*Capra hircus*), aging six to nine months, weighing 18 to 21 kg, being free of internal parasites, and appearing healthy, were used. These animals were maintained in an intensive system of management in nematode-free settings for one month before the experiment. On the basis of zero nematode fecal egg counts, the kids were randomly divided into four equal groups of five each (G1–G4). Each animal in (G1), (G3), and (G4) received 10,000 infective L3 orally on the first day of the trial. Goats of (G1) (infected-untreated) were regarded as the control positive group. In the fifth week postinfection, goats of (G3) (infected-albendazole-treated) were treated orally with albendazole at a single dose of 5 mg/kg.BW (as a reference drug, EVA Pharma, Egypt) while goats of (G2) (uninfected-BA-EE-treated as control) and (G4) (infected-BA-EE-treated) were drenched orally with BA-EE at a single dosage of 9 g/kg.BW suspended in 250 mL distilled water. The trial lasted nine weeks. Each group was maintained in its own enclosure.

### Abomasum tissue sampling

In accordance with the recommendations of the ’Ethical Committee for Medical Research (ECMR) of the National Research Centre (NRC), the animals were slaughtered at the end of the experiment. The abomasum specimens were quickly removed, cleaned with 0.9% ice-cold NaCl, and then kept at − 80 °C until used to measure antioxidant/oxidant markers, gene expression of inflammatory cytokines, and apoptotic markers.

### Preparation of an abomasum homogenate

A homogenate of 10% (w/v) was produced by homogenizing one gram of abomasum tissue in an ice-cold 1.15% potassium chloride solution in a 50 mmol/L potassium phosphate buffer solution (pH 7.4). The homogenate was centrifuged at 4,000 *×g* for five minutes at 4 °C. The supernatant was taken and stored at − 80 °C until used to determine antioxidant/oxidant markers.

### Assessment antioxidant/oxidant markers in abomasum homogenate

Reduced glutathione (GSH) (Ellman 1959) and malondialdehyde (MDA) (Ohkawa et al. 1979) concentrations as well as the superoxide dismutase (SOD) (Marklund and Marklund 1974) activity were measured in the supernatant of the abomasum homogenate using a kit purchased from Bio-diagnostic Co., Egypt. Activities of glutathione-S-transferase (GST) (Habig et al. 1974) and catalase (CAT) (Aebi 1984) were chemically estimated. All analytical chemicals used were obtained from Sigma-Aldrich, USA. All parameters were measured using a spectrophotometer (T80 UV-Vis, PG Instruments Ltd., UK).

### RNA extraction and quantitative real-time PCR

Total RNA was extracted from the abomasum tissue using TRIzol (Invitrogen, Carlsbad, CA, USA) in accordance with the manufacturer’s instructions. The yield and quality of the RNA were analyzed using a NanoDrop™ 1000 spectrophotometer and gel electrophoresis (Thermo Fisher Scientific, USA). RNA (1 µg) was treated with an RNase-free DNase kit (Promega) to remove any genomic DNA contamination, and cDNA was synthesized using a reverse kit (RT-PreMix Kit for cDNA synthesis, iNtRON Biotechnology, Seongnam-Si, Korea). The reaction was carried out in a thermal cycler under specific cycling conditions: 42 °C for 60 min and 90 °C for 5 min.

### Real-time PCR analysis

In the present study, cytokine genes interleukin 3 (IL-3), IL-6, IL-10, and tumor necrosis factor alpha (TNF-α) as well as apoptotic genes proapoptotic Bax (B-cell lymphoma-2 protein-associated X protein) and antiapoptotic Bcl-2 (B-cell lymphoma-2 protein) were used. β-actin and glyceraldehyde-3-phosphate dehydrogenase (GAPDH) were used as housekeeping genes. Table 1 lists the properties of primers. A real-time polymerase chain reaction (real-time PCR) was carried out using a Stratagene Mx3005P real-time PCR System (Agilent Technologies) in a 20 µL reaction. Each 20 µL PCR cocktail contained 1 µL cDNA, 10 µL TOPreal™ qPCR 2X PreMix (SYBR Green with low ROX; Enzynomics), 0.75 µL forward primer (10 pmol), 0.75 µL reverse primer (10 pmol), and 7.5 µL ddH_2_O. Amplification conditions included 15 min at 95 °C, followed by 40 cycles at 95 °C for 15 s, 58–63 °C for 15 s, and 72 °C for 30 s. Melting-curve analysis was conducted after each real-time PCR. Gene expression data were normalized to β-actin and GAPDH and analyzed using the 2^−ΔΔCt^ method (Livak and Schmittgen 2001):

**Table 1:** Primers information for the measurement of mRNA expression by a quantitative reverse-transcriptase polymerase chain reaction

Gene Name	Sequence 5’ to 3’	Product length (bp)	Accession no.	References
IL-3	F: ACCTCCTTCTGCTCCTGCTT R: TATTCCCAAGTCCCCATCTT	193	Z18897	Smeed et al. (2007)
IL-6	F: TCCAGAACGAGTTTGAGG R: CATCCGAATAGCTCTCAG	236	NM_001285640.1	Smeed et al. (2007)
	F: CTGTTGACCCAGTCTCTGCT R: ACCGCCTTTGCTCTTGTTT	305	U11421	Smeed et al. (2007)
TNF-α	F: ACAACGGGCCACCAACCATCR: TCTCCCAGGACACCTTGACC	360	X14828.1	Smeed et al. (2007)
Bax	F: GCATCCACCAAGAAGCTGAG-3′ R: CCGCCACTCGGAAAAAGAC-3′	130	NM_173894.1	Veshkini et al. (2018)
Bcl-2	F: ATGTGTGTGGAGAGCGTCA-3′ R: AGAGACAGCCAGGAGAAATC-3′	182	NM_001166486.1	Veshkini et al. (2018)
GAPDH	F: GTCTTCACTACCATGGAGAAGG R: TCATGGATGACCTTGGCCAG	197	BC102589.1	Finot et al. (2011)
β-actin	F: TGGGCATGGAATCCTG R: GGCGCGATGATCTTGAT	196	NM_001314342.1	Puech et al. (2015)

IL: Interleukin. TNF-α: Tumor necrosis factor alpha. Bax: B-cell lymphoma-2 protein-associated X protein

Bcl-2: B-cell lymphoma-2 protein. GAPDH: Glyceraldehyde-3- phosphate dehydrogenase

ΔΔCt = (Ct, target gene − Ct, housekeeping genes) infected samples − (Ct, target gene − Ct, housekeeping genes) control samples, where Ct is the cycle threshold.

The data are represented using the 2^−ΔΔCt^ method as the fold change in target gene expression normalized to the housekeeping genes β-actin and GAPDH to normalize input RNA quality, RNA quantity, and reverse transcription efficiency.

### Statistical analysis

All data were statistically analyzed and presented as means ± standard error (SE). Differences between data of antioxidant/oxidant parameters in different groups were tested for significance by the one-way analysis of variance (ANOVA) followed by Duncan’s multiple range test using the SPSS version 17 (SPSS Inc., Chicago, IL, USA) computer program. Statistical analyses of data of gene expressions of inflammatory cytokines and apoptosis were conducted with Prism v8.3.1 (GraphPad, La Jolla, CA, USA). Differences between groups were evaluated by one-way ANOVA, and the significant level was set as *P ≤* 0.05.

## Results

### Antioxidant/oxidant status

The data presented in Table 2 illustrate the effect of BA-EE and albendazole (as a standard drug) on the levels of oxidant and antioxidant parameters in the abomasum homogenate of goats experimentally infected with *H. contortus.*

**Table 2:** Effect of *Balanites aegyptiaca* fruit ethanol extract (BA-EE) on antioxidant/oxidant markers in the abomasum tissue homogenates of goats experimentally infected with *Haemonchus contortus*. (Mean ± SE, *n* = 5)

Groups	GSH (µmol/mg protein)	GST (nmol/min/mg protein)	SOD (U/mg protein)	CAT (µmol of H_2_O_2_ /min/mg protein)	MDA (nmol/mg protein)
Infected-untreated	1.62 ± 0.03^a^	0.13 ± 0.01^a^	21.07 ± 0.50^a^	0.05 ± 0.00^a^	53.36 ± 1.95^d^
Uninfected-BA-EE-treated	2.82 ± 0.09^c^	0.23 ± 0.01^b^	28.63 ± 1.80^b^	0.05 ± 0.00^a^	16.07 ± 1.67^a^
Infected-Albendazole-treated	2.03 ± 0.00^b^	0.23 ± 0.02^b^	26.41 ± 1.38^b^	0.05 ± 0.00^a^	41.55 ± 1.45^c^
Infected-BA-EE-treated	3.00 ± 0.18^c^	0.35 ± 0.02^c^	56.47 ± 1.66^c^	0.07 ± 0.00^b^	29.45 ± 1.19^b^

Means with different letters in the same column are significantly different at *P* < 0.05.GSH: Reduced glutathione

GST: Glutathione-S-transferase. SOD: Superoxide dismutase. CAT: Catalase. MDA: Malondialdehyde

Goats infected with *H. contortus* and untreated revealed that GSH content and GST and SOD activities significantly (*P <* 0.001) decreased while the MDA level markedly (*P <* 0.001) increased in the abomasum homogenate compared with the other groups. After BA-EE and albendazole treatment, the GSH content and SOD and GST activities markedly increased while the level of MDA significantly decreased in comparison with the infected-untreated goats. After treatment with BA-EE, CAT markedly (*P <* 0.05) elevated in comparison with the other groups. There were significant differences between all parameters’ values after treatment with BA-EE and albendazole, which revealed that BA-EE had greater antioxidant effect than albendazole (Table 2**)**.

### Cytokine gene expressions

A quantitative PCR was carried out to estimate the changes in mRNA gene expressions of cytokines (IL-3, IL-6, IL-10, and TNF-α) in the abomasum tissue of the infected goats compared with the other groups, and β-actin and GAPDH were used as housekeeping genes. All of the tested genes showed a single peak in the PCR melting curve, indicating the specificity of amplification and the lack of primer-dimer formation during the reaction. Each sample’s quantitative gene expression was estimated, and the Ct means were calculated.

**Fig. 1:**
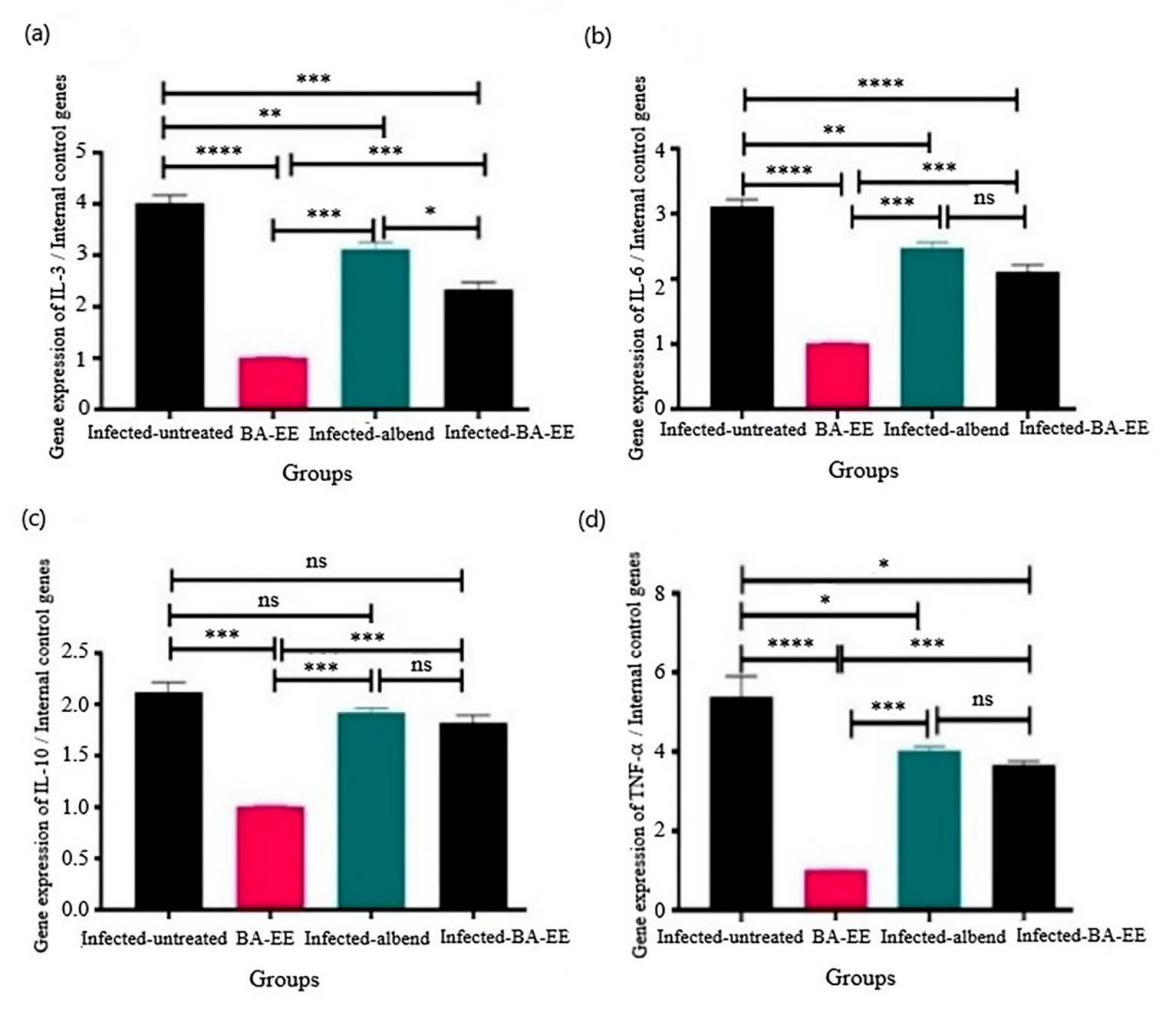
Effect of *Balanites aegyptiaca* fruit ethanol extract (BA-EE) on fold changes in mRNA gene expression of cytokines: (**a**) IL-3, (**b**) IL-6, (**c**) IL-10, and (**d**) TNF-α in the abomasum tissue of goats experimentally infected with *Haemonchus contortus*. The results are expressed after normalization (ratio) with two housekeeping genes (GAPDH and β-actin) Bars with asterisks (*) denote a significant difference in the expression levels between groups: ^*^P ≤ 0.05, ^**^P ≤ 0.01, ^***^P ≤ 0.001, ^****^P ≤ 0.0001; ns: nonsignificant, *P* > 0.05] IL: interleukin. TNF-α: tumor necrosis factor alpha. albend: albendazole

IL-3 is a pleiotropic hematopoietic cytokine. Its quantitative mRNA gene expression was significantly (*P ≤* 0.0001) upregulated in the infected-untreated goats compared with the uninfected-BA-EE-treated ones. Treatment with albendazole or BA-EE markedly downregulated the mRNA gene expression levels, with a significant difference between their values (− 3.1- and − 2.3-fold change; *P =* 0.005, *P ≤* 0.000; respectively; Fig. 1a).

The cytokine IL-6 has a pleotropic role in controlling inflammation and the immune system. The relative mRNA gene expression of IL-6 was markedly (*P =* 0.0003) upregulated in the infected-untreated goats compared with the uninfected-BA-EE-treated ones. Furthermore, treatment with either albendazole or BA-EE downregulated the mRNA gene expression levels, with no significant difference between their values (− 2.5- and − 2.1-fold change; *P =* 0.004, *P =* 0.0002) in the infected-albendazole-treated and infected-BA-EE-treated groups, respectively, in comparison with the infected-untreated group (Fig. 1b).

The cytokine IL-10, which has strong anti-inflammatory properties, is essential for limiting the response of the host immune system’ to pathogens. The relative mRNA gene expression of IL-10 was significantly (*P =* 0.001) upregulated in the infected-untreated goats compared with the uninfected-BA-EE-treated ones, whereas treatment with either BA-EE or albendazole reduced fold expression with no significant difference (*P =* 0.788; Fig. 1c).

The pro-inflammatory TNF-α cytokine is released by macrophages and regulates several cellular processes, such as cell division, proliferation, apoptosis, lipid metabolism, and coagulation. Gene expression results indicated a significant (*P ≤* 0.0001) fold increase in the infected-untreated group compared with the uninfected-BA-EE-treated one. Meanwhile, treatment with either BA-EE or albendazole decreased fold expression, with no significant difference between their values (*P =* 0.785; Fig. 1d).

### Apoptotic gene expression

**Fig. 2:**
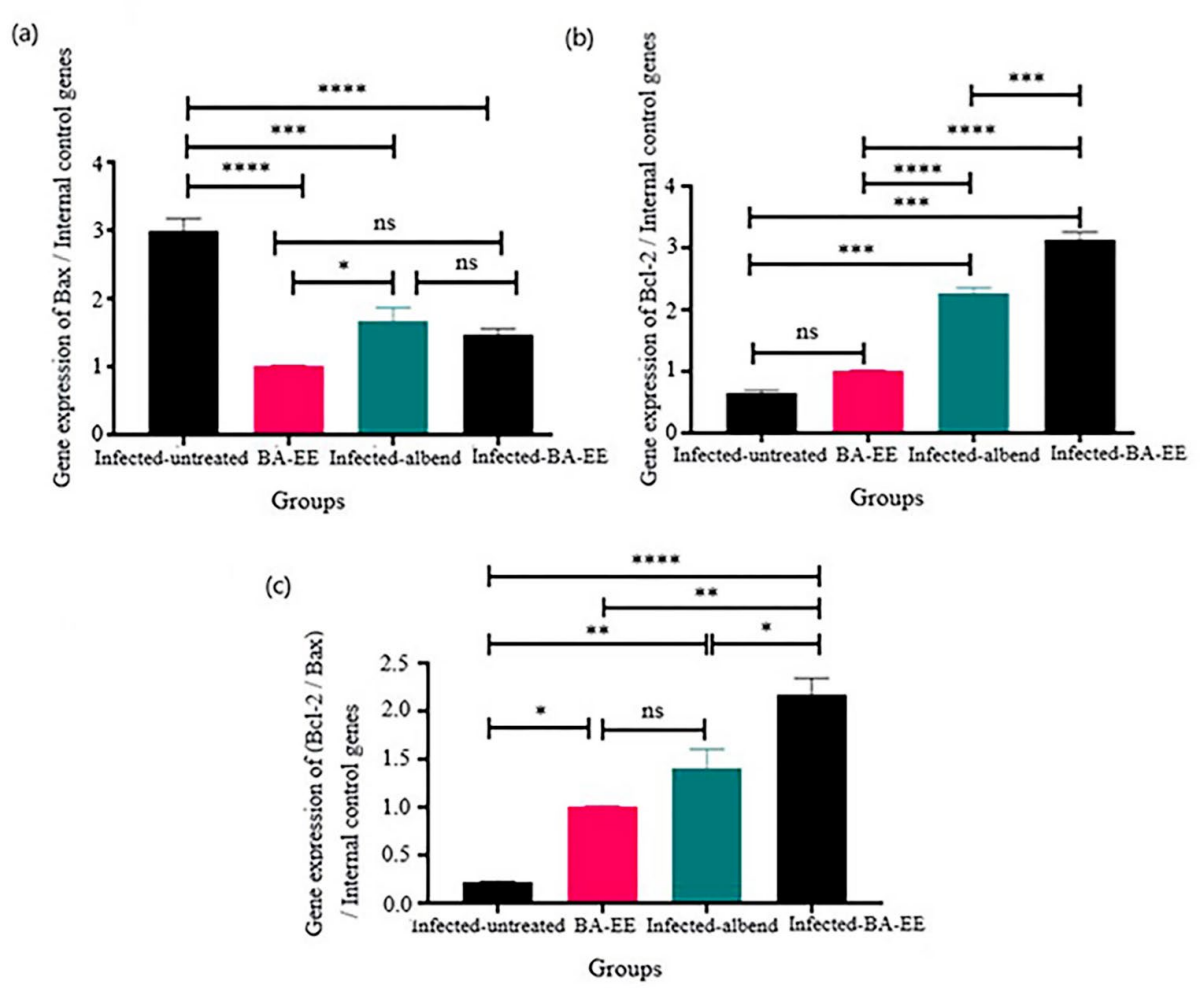
Effect of *Balanites aegyptiaca* fruit ethanol extract (BA-EE) on fold changes in mRNA apoptotic gene expression: (**a**) Bax (proapoptotic), (**b**) Bcl-2 (antiapoptotic), and (**c**) the ratio of Bcl-2 to Bax gene expression in the abomasum tissue of goats experimentally infected with *Haemonchus contortus*. The results are expressed after normalization (ratio) with two housekeeping genes (GAPDH and β-actin) Bars with asterisks (*) denote a significant difference in the expression levels between groups: ^*^P ≤ 0.05, ^**^P ≤ 0.01, ^***^P ≤ 0.001, ^****^P ≤ 0.0001; ns: nonsignificant, *P* > 0.05. Bax: B-cell lymphoma-2 protein-associated X protein. Bcl-2: B-cell lymphoma-2 protein. albend: albendazole

The quantitative gene expression of proapoptotic Bax was significantly (*P ≤* 0.0001) upregulated in the infected-untreated goats compared with the uninfected-BA-EE-treated ones. Furthermore, treatment with either albendazole or BA-EE markedly downregulated the expression levels (− 1.7- and − 1.5-fold change; *P =* 0.0007, *P =* 0.0003, respectively). There was no statistically significant difference between the infected-BA-EE-treated and uninfected-BA-EE-treated groups (*P =* 0.167). Also, there was no significant change (*P =* 0.752) in Bax expression in the infected-albendazole-treated or infected-BA-EE-treated group (Fig. 2a).

The expression of the antiapoptotic gene Bcl-2 in the infected-untreated group showed a downregulation that was nonsignificant compared with that in the uninfected-BA-EE-treated group. Furthermore, treatment with either albendazole or BA-EE upregulated the expression levels (2.3- and 3.1-fold change; *P =* 0.0003, *P =* 0.0001, respectively; Fig. 2b).

#### Bcl-2/Bax ratio

In terms of the expressions of the Bax and Bcl-2 genes, it was found that the capacity of antiapoptotic Bcl-2 to suppress the activity of proapoptotic Bax was impacted by both the expression levels of these genes and the ratio of Bcl-2 to Bax. The mRNA content of Bax in the abomasum tissue of the infected-BA-EE-treated goats dropped by 47% compared with the infected-untreated group (*P =* 0.0001; Fig. 2a). Moreover, we found that Bcl-2 gene expression increased by 21% in the abomasum tissue of infected-BA-EE-treated goats compared with the infected-untreated ones (*P =* 0.0001; Fig. 2b). Additionally, the ratio of Bcl-2 to Bax in the abomasa of the infected-untreated goats (21%) was considerably lower (*P =* 0.0001) than that in the uninfected-BA-EE-treated ones (Fig. 2c).

## Discussion

Because of the highly anthelmintic efficacy of BF-EE in our previous study (Jaheed et al. 2019), a maximal reduction in eggs per gram of feces (EPG) (88.10%) and worm burden (94.66%) was recorded in the fourth week posttreatment in contrast to the efficacy of albendazole (98.29% and 96.95%, respectively). Also, BF-EE improved the health condition and metabolic profile of infected goats. In our previous work (Jaheed et al. 2019), the phytochemical components screened included saponins, alkaloids, flavonoids, phenolics, and terpenoids. In addition, the crude BA-EE exhibited a strong in vitro total antioxidant activity (2128.86 mg/100 g ascorbic acid equivalent).

MDA, as a lipid peroxidation by-product, is regarded as a good indicator for cell damage, which confirmed the increased release of free radicals in the *H. contortus*–infected animals (Ayala et al. 2014). MDA was markedly elevated in the abomasum tissue after *H. contortus* infection (Jaheed et al. 2020; Mravčáková et al. 2021), which is consistent with the present result. Reduced GSH (γ-L-glutamyl-L-cysteinyl-glycine) is a tripeptide thiol that helps in eliminating the cytotoxic effect of ROS and protecting cells from it. *H. contortus* infection was accompanied by a reduction in the levels of GSH in the abomasum tissue, which were comparable to those reported by Rashid and Irshadullah (2019) and Ahmed et al. (2019). According to the hematophagous activity of both adult and fourth larval stage of *H. contortus*, the decreased GSH level could be due to a decrease in the precursor of the amino acid levels (cysteine and cystine) responsible for GSH synthesis (Sido et al. 1998). Moreover, the antioxidant function of GSH is accomplished largely by using the GSH as a substrate for GPx, which detoxifies peroxides to water, while two GSH molecules are oxidized to form oxidized GSH (GSSG). So, the reduced level of GSH may also be explained by the increased oxidation of GSH to GSSG, catalyzed by free radicals (Morris et al. 2013). Similar findings have been reported in mice infected with *Eimeria papillata* (Abdel-Tawab et al. 2020) and *Hymenolepis nana* (Abdel-Latif et al. 2017).

GSTs are a protein family that catalyzes the conjugation of GSH to various electrophiles via the sulfur atom of cysteine (Salinas and Wong 1999). Similarly, we found that *H. contortus* infection caused a decreased GST activity in the abomasum tissue, which is somewhat consistent with the observation made by Bártíková et al. (2010), who showed that intestinal GST activity was not significantly decreased after sheep suffered from haemonchosis.

Also, a significant decrease in hepatic GST activity was observed in male mouflons (Skálová et al. 2007) and Mouflon ewes infected with *Dicrocoelium dendriticum* (Křížová et al. 2008) and in the ileum of rats infected with *Cryptosporidium* (Abd El-Aziz et al. 2014). In contrast, an increase in GST activity was observed in goats spontaneously infected with haemonchosis (Rashid and Irshadullah 2019). The reason for the different influence of *H. contortus* infection on the GST activity may be the worm burden as reported by Rashid and Irshadullah (2019), who studied the effect of infection in naturally infected goats.

On the other hand, the infected abomasum tissue showed a significant decline in SOD activity, which is consistent with the findings of Rashid and Irshadullah (2014), Ahmed et al. (2019), and Alam et al. (2020). Similarly, the lower SOD activity has been reported in ovine naturally infected with *Fasciola hepatica* (Kolodziejczyk et al. 2005), *Theilaria* sp. (Nazifi et al. 2011) and *Babesia ovis* (Esmaeilnejad et al. 2012). The inhibition of SOD activity in the infected abomasum tissue could be attributed to the overproduction of the superoxide anion which causes inactivation and depletion of SOD storage (Rashid and Irshadullah 2014). Generally, these findings indicated an enhanced oxidative stress with a decline in antioxidant defense in the *H. contortus*–infected goats.

Cytokines with pro- and anti-inflammatory actions are produced by lymphocytes or monocytes. According to Bohstam et al. (2017), the balance between pro-inflammatory cytokines (IL-1β, IL-2, Il-6, TNF-α, IFN-γ and IL-8) and anti-inflammatory cytokines (TGFβ, IL-4 and IL-10) is considered as a crucial factor in immunological reaction homeostasis and inflammation, which underpins numerous diseases. According to Gossner et al. (2013) and Estrada-Reyes et al. (2015), haemonchosis triggers significant alterations in the production of TH1 (IL-2 and IL-8) and TH2 (IL-4, IL-5, IL-6, IL-13 and IL-10) cytokines, either in peripheral blood or in abomasum wall. It was documented that the *H. contortus* ES products significantly affect the host’s immune responses to parasitic infection, which are primarily cytokine production and apoptosis (Sun et al. 2007; Ehsan et al. 2017, 2020).

In the present study, haemonchosis elevated IL-3, IL-6, IL-10, and TNF-α gene expressions in abomasum tissue mRNA, which was attributed to the activation of the inflammatory cascade triggered by infection. *H. contortus* infection increases pro-inflammatory cytokines because of the elevated intracellular ROS in the abomasum, which in turn stimulates the transcription factor nuclear factor kappa B (NF-κB) (Bąska and Norbury 2022). NF-κB activation triggers inflammatory signaling cascades and an increase in TNF-α expression in the abomasum (Toscano et al. 2019). TNF-α stimulates the production of inflammatory cytokines, particularly IL-3, IL-6, and IL-10 (Jang et al. 2021; Kany et al. 2019). These results agree with Terefe et al. (2007) which identified increased levels of IL-3 gene expressions in *H. contortus*–infected lambs. Meanwhile, Buendía-Jiménez et al. (2015) revealed increased IL-6 gene expression in sheep infected with *H. contortus*. Moreover, the same results were noticed in the tropical sheep breed Pelibuey infected with *H. contortus* (Estrada-Reyes et al. 2015, 2017).

Although apoptosis is a form of programmed cell death that is essential for proper tissue development, a variety of diseases including helminths are able to manipulate host apoptosis pathways to their benefit (Donskow-Schmelter and Doligalska 2005). Apoptotic proteins Bcl-2 and Bax govern cell death. Bcl-2 pore-stabilizing proteins maintain mitochondrial membrane barrier function, inhibiting apoptosis and prolonging cell survival. Bax is primarily found in cytoplasm where it functions as a pore-destabilizing protein competing with the apoptotic factor Bcl-2. Apoptosis is regulated by the balance between Bax and Bcl-2 expression (You et al. 2019; Zheng and Kanneganti 2020).

The current results of apoptotic gene expression patterns revealed higher levels of Bax, decreased levels of Bcl-2, and changes in the ratio of Bcl-2 to Bax in the infected abomasum tissue. Accumulating evidence suggests that the infected abomasum tissue is mediated by apoptotic signaling pathways, which are also connected to ROS-induced oxidative damage (Sies et al. 2022). This is supported by our findings that *H. contortus* causes an increase in the MDA levels and depletion in the antioxidant state of the infected animals, which is represented by lowered GSH, GST, and SOD levels. Lipid peroxidation, a process that directly degrades phospholipids, is utilized as a cell-death signal to induce programmed cell death (Lennicke and Cochemé 2021).

Furthermore, the recombinant galectins of the *H. contortus* parasite (rHco-gal-m/f proteins) were shown to trigger apoptosis in goat peripheral blood lymphocytes (PBLCs) (Sun et al. 2007). Ehsan et al. (2017; 2020) suggested that two key ES proteins of *H. contortus*, arginine kinase and elongation factor 1 alpha (rHcEF-1α), inhibited the proliferation and enhanced the apoptosis of goat peripheral blood mononuclear cells (PBMCs) *in vitro.* According to La Sala et al. (2001), arginine kinase controls the transformation of adenosine triphosphate (ATP) molecules into arginine, which is necessary for metabolic and cellular processes such as cytokine production, cellular response, plasma membrane alteration, and apoptosis.

In the present study, the infected goats treated with BA-EE showed an increase in the antioxidant levels (GSH, SOD, GST, and CAT) accompanied by a decrease in the MDA level. This could be because crude BA-EE exhibited a strong antioxidant activity in vitro (Jaheed et al. 2019). Furthermore, BA-EE reduced the production of pro-inflammatory cytokines, including TNF-α, IL-3, and IL-6; strengthened its anti-inflammatory role (Gaur et al. 2008; Elkareem et al. 2021); and regulated apoptosis through the balance between Bax and Bcl-2, the two markers of apoptosis gene expression. This could be due to either the antioxidant activity (Jaheed et al. 2019; Sedky et al. 2022) or the anthelminthic efficacy of BA-EE which caused the reduction of worm burden (Jaheed et al. 2019; Shalaby et al. 2020).

As was previously mentioned, the anti-inflammatory properties of BA-EE may be responsible for its inhibition of the release of the inflammatory mediators IL-6 and TNF-α with enhancement of production of the anti-inflammatory IL-10, as well as antioxidant properties of BA-EE (Gaur et al. 2008; Elkareem et al. 2021).

*B. aegyptiaca* fruit extracts have been shown to contain phytochemical components such as phenolic compounds (gentisic, *p*-coumaric, caffeic, ferulic, and sinapic acids), quinones, flavonoids, flavones, tannins, coumarins, terpenoids, essential oils, and alkaloids (Jaheed et al. 2019; Murthy et al. 2021; Sedky et al. 2022). Condensed tannins are the most important constituent of *B. aegyptiaca* and have an anthelminthic activity against *H. contortus in vitro* (Assefa et al. 2018). It has been shown that both ethanolic and aqueous extracts of *B. aegyptiaca* fruits exhibit significant antioxidant power in vitro (Jaheed et al. 2019; Sedky et al. 2022). The antioxidant and anti-inflammatory properties of *B. aegyptiaca* have been linked to its phenolic and flavonoid content (Yahfoufi et al. 2018; Murthy et al. 2021). Natural compounds, with their ability to modulate pro-inflammatory gene expression and antioxidant properties such as ROS scavenging, play a crucial role in regulating inflammatory signaling (Malireddy et al. 2012; Yahfoufi et al. 2018). These findings could be attributed to the bioactive BA-EE compounds, which induce different parasiticidal mechanisms. Previous publications have shown that BA-EE has promising anthelmintic efficacy (Jaheed et al. 2019; Shalaby et al. 2016, 2020; Hassan et al. 2021).

Studies conducted in vivo and in vitro have shown that polyphenols have an impact on macrophages by suppressing a number of essential inflammatory response regulators, such as TNF-α, IL-1, and IL-6 (González et al. 2011). Additionally, flavonoids have been demonstrated to reduce the levels of a variety of pro-inflammatory cytokines, particularly IL-1, IL-6, IL-8, and TNF-α (Comalada et al. 2006).

Similarly, several polyphenol analogs, such as the curcumin analog EF31, have been demonstrated to decrease the secretion and expression of IL-1, IL-6, and TNF-α in mice (Olivera et al. 2012). Likewise, TNF-α and IL-6 production is reduced in THP1 macrophages by willow bark, meadowsweet, and extracts of chamomile and isolated polyphenols such as quercetin found in these extracts (Drummond et al. 2013). Also, the phytochemical analysis of BA-EE revealed the presence of α-linolenic acid as one of the major constituents. α-Linolenic acid is an omega-3 fatty acid known to possess an anti-inflammatory activity (Otuechere and Farombi, 2020), which may explain the reported inhibition of the pro-inflammatory mediators.

The treatment with albendazole enhanced the host immune response by changing the cellular infiltration and thus altering the cellular immunity (Ricken et al. 2017). Albendazole treatment in goats reduced *H. contortus* infection (87.9%) more than levamisole (80.2%) and parbendazole (83.9%) did (Charles et al. 1989). Administration of albendazole influenced the balance of immune response and promoted the secretion of pro-inflammatory factors, which is beneficial to parasite clearance (Wu et al. 2021). Albendazole treatment supported the activation of the host immune system by reducing the immunosuppressive functions of the parasite. It also induced degenerative alterations in the parasite tegument and the cells damaging its metabolism, resulting in immobilization and death of the cyst (Jung-Cook 2012).

In conclusion, as a promising supplement, BA-EE has an anthelmintic effect exhibited by its protective role in the abomasum tissue of goats infected with *H. contortus*. It exhibited modulatory effects on oxidant/antioxidant profile and inflammatory cytokines, as well as up- and downregulatory effects on the mRNA gene expression levels of antiapoptotic and proapoptotic genes, respectively. This could be explained via the antioxidant and anti-inflammatory activities of its bioactive ingredients.

## Data Availability

The data that support the findings of this study are available from the corresponding author upon reasonable request.

## References

[CR1] Abd El-Aziz TH, El-Beih NM, Soufy H et al (2014) Effect of Egyptian propolis on lipid profile and oxidative status in comparison with nitazoxanide in immunosuppressed rats infected with *Cryptosporidium spp*. Global Veterinaria 13:17–27

[CR2] Abdel-Latif M, El‐Shahawi G, Aboelhadid SM et al (2017) Immunoprotective effect of chitosan particles on *Hymenolepis nana*–infected mice. Scand J Immunol 86:83–90 28513991 10.1111/sji.12568

[CR3] Abdel-Tawab H, Abdel-Haleem HM, Abdel-Baki AAS et al (2020) Anticoccidial and antioxidant activities of *Moringa oleifera* leaf extract on murine intestinal eimeriosis. Acta Parasitol 65:823–830 32472400 10.2478/s11686-020-00219-w

[CR4] Aebi H (1984) Catalase in vitro. Methods Enzymol 105:121–126 6727660 10.1016/S0076-6879(84)05016-3

[CR5] Ahmed WM, Mostafa AM, Al-Salahy MB et al (2019) The potential effect of *Allium sativum* and *Coriandrum Sativum* as natural sources of antioxidants on *Haemonchus contortus* infection in sheep. Egypt Veter Med Soc Parasitol J 15:89–113

[CR6] Alam RT, Hassanen EA, El-Mandrawy SA (2020) *Heamonchus Contortus* infection in Sheep and Goats: alterations in haematological, biochemical, immunological, trace element and oxidative stress markers. J Appl Animal Res 48:357–364 10.1080/09712119.2020.1802281

[CR7] Albadawi ROE (2010) In vivo and in vitro anthelmintic activity of *Balanites aegyptiaca* and *Artemisia herba Alba* on *Haemonchu*s *contortus* of sheep. Ph.D. Faculty of Veterinary Science, University of Khartoum, Sudan

[CR8] Arsenopoulos V, Fthenakis GC, Eleni I et al (2021) Haemonchosis: a challenging parasitic infection of sheep and goats konstantinos. Animals 11:363 33535656 10.3390/ani11020363 PMC7912824

[CR9] Assefa A, Kechero Y, Tolemariam T et al (2018) Anthelmintic effects of indigenous multipurpose fodder tree extracts against *Haemonchus contortus*. Trop Animal Health Prod 50:727–732 29235047 10.1007/s11250-017-1488-0

[CR10] Ayala A, Munoz MF, Arguelles S (2014) Lipid peroxidation: production, metabolism, and signaling mechanisms of malondialdehyde and 4-hydroxy-2-nonenal. Oxidat Med Cell Long 2014:360438 10.1155/2014/360438 PMC4066722 24999379

[CR11] Bártíková H, Křížová V, Lamka J et al (2010) Flubendazole metabolism and biotransformation enzymes activities in healthy sheep and sheep with haemonchosis. J Veter Pharmacol Therap 33:56–62 20444026 10.1111/j.1365-2885.2009.01112.x

[CR12] Bąska P, Norbury LJ (2022) The role of nuclear factor kappa B (NF-κB) in the immune response against parasites. Pathogens 11:310 35335634 10.3390/pathogens11030310 PMC8950322

[CR13] Besier RB, Kahn LP, Sargison ND et al (2016) The pathophysiology, ecology and epidemiology of *Haemonchus contortus* infection in small ruminants. Adv Parasitol 93:95–143 27238004 10.1016/bs.apar.2016.02.022

[CR14] Bihaqi SJ, Allaie IM, Banday MAA et al (2020) Multiple anthelmintic resistance in gastrointestinal nematodes of Caprines on Mountain Research Centre for sheep and goat at Kashmir Valley, India. Paras Epidemiol Control 11:e00163 32984565 10.1016/j.parepi.2020.e00163 PMC7494505

[CR15] Bohstam M, Asgary S, Kouhpayeh S et al (2017) Aptamers against pro- and anti-inflammatory cytokines, a review. Inflammation 40:340–349 27878687 10.1007/s10753-016-0477-1

[CR16] Buendía-Jiménez JA, Muñoz-Guzmán MA, Vega-López MA et al (2015) Partial protection and abomasal cytokine expression in sheep experimentally infected with *Haemonchus contortus* and pre-treated with *Taenia hydatigena* vesicular concentrate. Veter Parasitol 211:60–66 25959643 10.1016/j.vetpar.2015.04.019

[CR17] Charles TP, Pompeu J, Miranda DB (1989) Efficacy of three broad-spectrum anthelmintics against gastrointestinal nematode infections of goats. Veter Parasitol 34(1–2):71–75 2588471 10.1016/0304-4017(89)90166-0

[CR18] Comalada M, Ballester I, Bailon E et al (2006) Inhibition of pro-inflammatory markers in primary bone marrow-derived mouse macrophages by naturally occurring flavonoids: Analysis of the structure-activity relationship. Biochem Pharmacol 72:1010–1021 16934226 10.1016/j.bcp.2006.07.016

[CR19] Donskow-Schmelter K, Doligalska M (2005) Apoptosis, a protective mechanism for pathogens and their hosts. Wiadomości Parazytologiczne 51:271–280 16913499

[CR20] Drummond EM, Harbourne N, Marete E et al (2013) Inhibition of proinflammatory biomarkers in THP1 macrophages by polyphenols derived from chamomile, meadowsweet and willow bark. Phytother Res 27:588–594 22711544 10.1002/ptr.4753

[CR21] Ehsan M, Gao W, Gadahi JA et al (2017) Arginine kinase from *Haemonchus contortus* decreased the proliferation and increased the apoptosis of goat PBMCs in vitro. Paras Vectors 10:311 10.1186/s13071-017-2244-z PMC5485575 28651566

[CR22] Ehsan M, Gadahi JA, Lu M et al (2020) Recombinant elongation factor 1 alpha of *Haemonchus contortus* affects the functions of goat PBMCs. Paras Immunol 42:e12703 32043596 10.1111/pim.12703 PMC7187238

[CR25] El-Newary SA, Ismail RF, Shaffie N et al (2016) Hepatoprotective, therapeutic and *in vivo* antioxidant activities of *Tagetes lucida* leaves alcoholic extract against paracetamol-induced hepatotoxicity rats. Int J Pharm Tech Res 9:327–341

[CR23] Elkareem HFA, Essawy AI, Ashry M et al (2021) Ameliorating and anti-inflammatory role of Balanites aegyptiaca aqueous extract on Doxorubicin-induced hepatotoxicity in male Wistar rats. Egypt Pharm J 20(2):157–165 10.4103/epj.epj_2_21

[CR24] Ellman GL (1959) Tissue sulfhydryl groups. Arch Biochem Biophys 82:70–77 13650640 10.1016/0003-9861(59)90090-6

[CR26] Esmaeilnejad B, Tavassoli M, Asri-Rezaei S et al (2012) Evaluation of antioxidant status and oxidative stress in sheep naturally infected with *Babesia ovis*. Veter Parasitol 185:124–130 22030375 10.1016/j.vetpar.2011.10.001

[CR28] Estrada-Reyes ZM, López-Reyes AG, Lagunas-Martínez A et al (2015) Relative expression analysis of IL-5 and IL-6 genes in tropical sheep breed Pelibuey infected with *Haemonchus contortus*. Paras Immunol 37:446–452 26094646 10.1111/pim.12211

[CR27] Estrada-Reyes Z, López-Arellano ME, Torres-Acosta F et al (2017) Cytokine and antioxidant gene profiles from peripheral blood mononuclear cells of Pelibuey lambs after *Haemonchus. contortus* infection. Paras Immunol 39:e12427 10.1111/pim.12427 28345265

[CR29] Finot L, Marnet PG, Dessauge F (2011) Reference gene selection for quantitative real-time PCR normalization: Application in the caprine mammary gland. Small Rumin Res 95:20–26 10.1016/j.smallrumres.2010.08.008

[CR30] Gadahi JA, Yongqian B, Ehsan M et al (2016) *Haemonchus contortus* excretory and secretory proteins (HcESPs) suppress functions of goat PBMCs in vitro. Oncotarget 7:35670–35679 27229536 10.18632/oncotarget.9589 PMC5094953

[CR31] Gaur K, Nema RK, Kori ML et al (2008) Anti-inflammatory and analgesic activity of *Balanites aegyptiaca* in experimental animal models. Int J Green Pharm 2:214–217 10.4103/0973-8258.44735

[CR32] González R, Ballester I, López-Posadas R et al (2011) Effects of flavonoids and other polyphenols on inflammation. Crit Rev Food Sci Nutr 51:331–362 21432698 10.1080/10408390903584094

[CR33] Gossner A, Wilkie H, Joshi A et al (2013) Exploring the abomasal node transcriptome for genes associated with resistance to the sheep nematode *Teladorsargia circumcincta*. Veter Res 44:68 23927007 10.1186/1297-9716-44-68 PMC3751673

[CR34] Habig WH, Pabst MJ, Jakoby WB (1974) Glutathione-S-transferases, the first enzymatic step in mercapturic acid formation. J Biol Chem 249:7130–7139 4436300 10.1016/S0021-9258(19)42083-8

[CR35] Hassan NMF, Zaghawa AA, Abu-Elezz NMT et al (2021) Efficacy of some Egyptian native plant extracts against haemonchus contortus in vitro and in experimentally infected sheep along with the associated haematological and biochemical alterations. Bull Natl Res Centre 45:180. 10.1186/s42269-021-00636-5 10.1186/s42269-021-00636-5

[CR36] Hoste H, Torres-Acosta JFJ, Quijada J et al (2016) Interactions between nutrition and infections with *Haemonchus contortus* and related gastrointestinal nematodes in small ruminants. Adv Parasitol 93:239–351 27238007 10.1016/bs.apar.2016.02.025

[CR37] Howlader MMR, Capitan SS, Eduardo SL et al (1997) Effects of experimental *Haemonchus contortus* infection on red blood cells and white blood cells of growing goats. Asian Austr J Animal Sci 10:679–682 10.5713/ajas.1997.679

[CR38] Jaheed E, Mohamed AH, Hassan NMF et al (2019) Evaluation of the curative effect of *Balanites aegyptiaca* fruits ethanolic extract on haemonchosis experimentally induced in Egyptian Baladi goats: Phytoanalytical, parasitological and hematological studies. J Paras Dis 43:638–650 31749536 10.1007/s12639-019-01143-1 PMC6841779

[CR39] Jaheed E, Mohamed AH, Nasr SM et al (2020) Therapeutic effect of *Balanites aegyptiaca* fruit’s ethanol extract in Egyptian Baladi goats experimentally infected with *Haemonchus Contortus*: Blood serum biochemical, oxidative stress markers and pathological studies. Egypt J Veter Sci 51:119–136 10.21608/ejvs.2019.20398.1140

[CR40] Jang DI, Lee AH, Shin HY et al (2021) The role of tumor necrosis factor alpha (TNF-α) in autoimmune disease and current TNF-α inhibitors in therapeutics. Int J Mol Sci 22:2719 33800290 10.3390/ijms22052719 PMC7962638

[CR41] Jung-Cook H (2012) Pharmacokinetic variability of anthelmintics: Implications for the treatment of neurocysticercosis. Expert Rev Clin Pharmacol 5:21–30. 10.1586/ecp.11.72 22142156 10.1586/ecp.11.72

[CR42] Kany S, Vollrath JT, Relja B (2019) Cytokines in inflammatory disease. Int J Mol Sci 20:6008 31795299 10.3390/ijms20236008 PMC6929211

[CR43] Kaplan RM (2020) Biology, epidemiology, diagnosis, and management of anthelmintic resistance in gastrointestinal nematodes of livestock. Veter Clin North Am Food Animal Pract 36:17–30 32029182 10.1016/j.cvfa.2019.12.001

[CR45] Koko WS, Galal M, Khalid HS (2000) Fasciolicidal efficacy of *Albizia anthelmintica* and *Balanites aegyptiaca* compared with albendazole. J Ethnopharmacol 71:247–252 10904170 10.1016/S0378-8741(00)00172-0

[CR44] Koko WS, Abdallab HS, Galala M et al (2005) Evaluation of oral therapy on *Mansonial Schistosomiasis* using single dose of *Balanites aegyptiaca* fruits and praziquantel. Fitoterapia 76:30–34 15664459 10.1016/j.fitote.2004.08.003

[CR46] Kolodziejczyk L, Siemieniuk E, Skrzydlewska E (2005) Antioxidant potential of rat liver in experimental infection with *Fasciola hepatica*. Parasitol Res 96:367–372 15928904 10.1007/s00436-005-1377-8

[CR47] Kotze AC, Prichard RK (2016) Anthelmintic resistance in *Haemonchus contortus*. Adv Parasitol 93:397–428 27238009 10.1016/bs.apar.2016.02.012

[CR48] Křížová V, Lamka J, Szotáková B et al (2008) Dicrocoeliosis of old mouflon ewes – effect on biotransformation enzymes and metabolism of anthelmintics *in vitro*. Open Veter Sci J 2:23–31 10.2174/1874318808002010023

[CR49] La Sala A, Ferrari D, Corinti S (2001) Extracellular ATP induces a distorted maturation of dendritic cells and inhibits their capacity to initiate Th1 responses. J Immunol 166:1611–1617 10.4049/jimmunol.166.3.1611 11160202

[CR50] Lennicke C, Cochemé HM (2021) Redox metabolism: ROS as specific molecular regulators of cell signaling and function. Mol Cell 81:3691–3707 34547234 10.1016/j.molcel.2021.08.018

[CR51] Livak KJ, Schmittgen TD (2001) Analysis of relative gene expression data using real-time quantitative PCR and the 2(-Delta Delta C (T)) method. Methods 25:402–408 11846609 10.1006/meth.2001.1262

[CR52] Machín C, Corripio-Miyar Y, Hern´andez JN et al (2021) Cellular and humoral immune responses associated with protection in sheep vaccinated against *Teladorsagia circumcincta*. Veter Res 52:89 34134748 10.1186/s13567-021-00960-8 PMC8207578

[CR53] Malireddy S, Kotha SR, Secor JD et al (2012) Phytochemical antioxidants modulate mammalian cellular epigenome: Implications in health and disease. Antiox Redox Signal 17:327–339 22404530 10.1089/ars.2012.4600 PMC3353820

[CR54] Marklund S, Marklund G (1974) Involvement of the superoxide anion radical in the autoxidation of pyrogallol and a convenient assay for superoxide dismutase. Eur J Biochem 47:469–474 4215654 10.1111/j.1432-1033.1974.tb03714.x

[CR55] McRae KM, Stear MJ, Good B et al (2015) The host immune response to gastrointestinal nematode infection in sheep. Paras Immunol 37:605–613 26480845 10.1111/pim.12290 PMC4744952

[CR56] Meda RNT, Vlase L, Lamien-Meda A et al (2011) Identification and quantification of phenolic compounds from *Balanites aegyptiaca* (L.) Del (*Balanitaceae*) galls and leaves by HPLC-MS. Nat Prod Res 25:93–99 21246435 10.1080/14786419.2010.482933

[CR57] Morris D, Khurasany M, Nguyen T et al (2013) Glutathione and infection. Biochim Biophys Acta 1830:3329–3349 23089304 10.1016/j.bbagen.2012.10.012

[CR58] Mravčáková D, Sobczak-Filipiak M, Váradyová Z et al (2021) Effect of *Artemisia absinthium* and *Malva sylvestris* on antioxidant parameters and abomasal histopathology in lambs experimentally infected with *Haemonchus contortus*. Animals 11:462 33572477 10.3390/ani11020462 PMC7916408

[CR59] Murthy HN, Yadav GG, Dewir YH et al (2021) Phytochemicals and biological activity of desert date (*Balanites aegyptiaca* (L.) Delile). Plants 10:32 10.3390/plants10010032 PMC7823407 33375570

[CR60] Nazifi S, Razvi SM, Kianiamin P et al (2011) Evaluation of erythrocyte antioxidant mechanisms: antioxidant enzyme, lipid peroxidation, and serum trace elements associated with progressive anemia in ovine maligant theileriosis. Parasitol Res 109:275–281 21301875 10.1007/s00436-010-2248-5

[CR61] Ohkawa H, Ohishi N, Yagi K (1979) Assay for lipid peroxides in animal tissues by thiobarbituric acid reaction. Analy Biochem 95:351–358 36810 10.1016/0003-2697(79)90738-3

[CR62] Olivera A, Moore TW, Hu F et al (2012) Inhibition of the NF-KB signaling pathway by the curcumin analog, 3,5-Bis(2-Pyridinylmethylidene)-4-Piperidone (EF31): Anti-inflammatory and anti-cancer properties. Int Immunopharmacol 12:368–377 22197802 10.1016/j.intimp.2011.12.009 PMC3372981

[CR63] Otuechere CA, Farombi EO (2020) Pterocarpus mildbraedii (Harms) extract resolves propanil-induced hepatic injury via repression of inflammatory stress responses in Wistar rats. J Food Biochem 44(12):e13506. 10.1111/jfbc.13506 33047371 10.1111/jfbc.13506

[CR64] Puech C, Dedieu L, Chantal I et al (2015) Design and evaluation of a unique SYBR Green real-time RT-PCR assay for quantification of five major cytokines in cattle, sheep and goats. BMC Veter Res 11:65 25889787 10.1186/s12917-015-0382-0 PMC4369058

[CR65] Rashid S, Irshadullah M (2014) Partial characterization of superoxide dismutase activity in the Barber pole worm-*Haemonchus contortus* infecting *Capra hircus* and abomasal tissue extracts. Asian Pacific J Trop Biomed 4:718–724 10.12980/APJTB.4.2014APJTB-2014-0099

[CR66] Rashid S, Irshadullah M (2019) Evaluation of antioxidant and oxidant status of goats (*Capra aegagrus hircus*) naturally infected with *Haemonchus contortus*. J Helminthol 94:e36 10.1017/S0022149X19000117 30761971

[CR67] Ricken FJ, Nell J, Grüner B et al (2017) Albendazole increases the inflammatory response and the amount of Em2-positive small particles of Echinococcus multilocularis (spems) in human hepatic alveolar echinococcosis lesions. PLOS Negl Trop Dis 11(5):e0005636. 10.1371/journal.pntd.0005636 28542546 10.1371/journal.pntd.0005636 PMC5462468

[CR68] Salinas AE, Wong MG (1999) Glutathione S-transferases-a review. Curr Med Chem 6:279–309 10101214 10.2174/0929867306666220208213032

[CR69] Sedky D, Mohamed AM, Fouad R et al (2022) Assessment of phytochemical, antioxidant and antibacterial activity of *Balanites Aegyptiaca* and *Curcuma Longa* against some bacterial pathogens isolated from dairy cow infected with mastitis. Adv Animal Veter Sci 10:160–169

[CR71] Shalaby MA, Moghazy FM, Shalaby HA et al (2010) Effect of methanolic extract of *Balanites aegyptiaca* fruits on enteral and parenteral stages of *Trichinella spiralis* in rats. Parasitol Res 107:17–25 20349194 10.1007/s00436-010-1827-9

[CR72] Shalaby H, Nasr S, Farag T (2016) Tegumental effects of methanolic extract of *Balanites aegyptiaca* fruits on adult *Paramphistomum microbothrium* (Fischoeder 1901) under laboratory conditions. Iran J Parasitol 11:396–405 28127347 PMC5256058

[CR70] Shalaby HA, Hassan NMF, Nasr SM et al (2020) An anthelmintic assessment of *Balanites aegyptiaca* fruits on some multiple drug resistant gastrointestinal helminthes affecting sheep. Egypt J Veter Sci 51:93–103 10.21608/ejvs.2019.19195.1119

[CR73] Sido B, Hack V, Hochlehnert A et al (1998) Impairment of intestinal glutathione synthesis in patients with inflammatory bowel disease. Gut 42:485–492 9616308 10.1136/gut.42.4.485 PMC1727080

[CR74] Sies H, Belousov VV, Chandel NS et al (2022) Defening roles of specific reactive oxygen species (ROS) in cell biology and physiology. Nat Rev Mol Cell Biol 23:499–515 35190722 10.1038/s41580-022-00456-z

[CR75] Skálová L, Křížová V, Cvilink V et al (2007) Mouflon (*Ovis musimon*) dicrocoeliosis: effects of parasitosis on the activities of biotransformation enzymes and albendazole metabolism in liver. Veter Parasitol 146:254–262 17386978 10.1016/j.vetpar.2007.02.026

[CR76] Smeed JA, Watkins CA, Rhind SM et al (2007) Differential cytokine gene expression profiles in the three pathological forms of sheep paratuberculosis. BMC Veter Res 3:18 17697353 10.1186/1746-6148-3-18 PMC1994670

[CR77] Soulsby EJL (1982) Helminths, arthropods and protozoa of domesticated animals. 7th edn. Baille Tindall, London

[CR78] Speroni E, Cervellati R, Innocenti G et al (2005) Anti-inflammatory, anti-nociceptive and antioxidant activities of *Balanites aegyptiaca* (L.) Delile. J Ethnopharmacol 98:117–125 10.1016/j.jep.2005.01.007 15763372

[CR79] Sun Y, Yan R, Muleke CI et al (2007) Recombinant galectins of *Haemonchus contortus* parasite induces apoptosis in the peripheral blood lymphocytes of goat. Int J Peptide Res Therap 13:387–392 10.1007/s10989-006-9045-0

[CR80] Terefe G, Lacroux C, Andreoletti O et al (2007) Immune response to *Haemonchus contortus* infection in susceptible (INRA 401) and resistant (Barbados Black Belly) breeds of lambs. Paras Immunol 29:415–424 17650183 10.1111/j.1365-3024.2007.00958.x

[CR81] Toscano JHB, Okino CH, Dos Santos IB et al (2019) Innate immune responses associated with resistance against *Haemonchus contortus* in Morada Nova Sheep. J Immunol Res 2019:3562672 10.1155/2019/3562672 PMC6877983 31815153

[CR82] Veshkini A, Mohammadi-Sangcheshmeh A, Ghanem N et al (2018) Oocyte maturation with royal jelly increases embryo development and reduces apoptosis in goats. Animal Reprod 15:124–134 34122643 10.21451/1984-3143-2017-AR986 PMC8186877

[CR83] Wang B, Wang Y, Zhang J et al (2023) ROSinduced lipid peroxidation modulates cell death outcome: Mechanisms behind apoptosis, autophagy, and ferroptosis. Arch Toxicol 97:1439–1451 37127681 10.1007/s00204-023-03476-6

[CR84] Wu J, Ma H-Z, Apaer S et al (2021) Impact of albendazole on cytokine and chemokine response profiles in *Echinococcus multilocularis*-inoculated mice. BioMed Res Int 2021:6628814. 10.1155/2021/6628814 10.1155/2021/6628814 PMC8121589 34041299

[CR85] Xu D-P, Li Y, Meng X et al (2017) Natural antioxidants in foods and medicinal plants: Extraction, assessment and resources. Int J Mol Sci 18:96 28067795 10.3390/ijms18010096 PMC5297730

[CR86] Yahfoufi N, Alsadi N, Jambi M et al (2018) The immunomodulatory and anti-inflammatory role of polyphenols (Review). Nutrients 10:1618 30400131 10.3390/nu10111618 PMC6266803

[CR87] You L, Yang C, Du Y et al (2019) Matrine exerts hepatotoxic effects via the ROS-dependent mitochondrial apoptosis pathway and inhibition of Nrf2-mediated antioxidant response. Oxidat Med Cell Long 2019:1045345 10.1155/2019/1045345 PMC6815593 31737162

[CR88] Zheng M and Kanneganti TD (2020) The regulation of the Zbp1-Nlrp3 inflammasome and its implications in pyroptosis, apoptosis, and necroptosis (Panoptosis). Immunol Rev 297:26–38 32729116 10.1111/imr.12909 PMC7811275

